# eHealth-Integrated Psychosocial and Physical Interventions for Chronic Pain in Older Adults: Scoping Review

**DOI:** 10.2196/55366

**Published:** 2024-07-29

**Authors:** Annalisa De Lucia, Cinzia Perlini, Alessandro Chiarotto, Sara Pachera, Ilenia Pasini, Lidia Del Piccolo, Valeria Donisi

**Affiliations:** 1 Section of Clinical Psychology Department of Neuroscience, Biomedicine and Movement Sciences University of Verona Verona Italy; 2 Department of General Practice, Erasmus University Medical Center Rotterdam Netherlands; 3 Fondazione Casa di Riposo S Giuseppe Nonprofit Organization of Social Utility San Martino Buon Albergo (Verona) Italy

**Keywords:** chronic pain, older adults, eHealth, scoping review, psychological intervention, physical intervention, multimodal intervention, biopsychosocial model for chronic pain, self-management, mobile phone

## Abstract

**Background:**

Chronic noncancer pain (CNCP) is highly present among older adults, affecting their physical, psychological, and social functioning. A biopsychosocial multimodal approach to CNCP management is currently extensively suggested by international clinical practice guidelines. Recently, the growing development and application of eHealth within pain management has yielded encouraging results in terms of effectiveness and feasibility; however, its use among the older population remains underexamined.

**Objective:**

The overall aim of this scoping review was to systematically map existing literature about eHealth multimodal interventions (including both physical and psychosocial components) targeting older adults with CNCP.

**Methods:**

This review adhered to the JBI methodology, a protocol was a priori registered as a preprint on the medRxiv platform, and the results were reported according to the PRISMA-ScR (Preferred Reporting Items for Systematic Reviews and Meta-Analyses extension for Scoping Reviews) guidelines. Four electronic databases (PubMed, Cochrane Central Register of Controlled Trials, Web of Science, and PsycINFO) were systematically searched for relevant articles. Studies were included if they reported on multimodal interventions (including both physical and psychosocial components) delivered through any eHealth modality to an older population with any type of CNCP. Two reviewers selected the studies: first by screening titles and abstracts and second by screening full-text articles. The quality of the included studies was evaluated using the Quality Assessment Tool for Studies with Diverse Designs. The results of the studies were summarized narratively.

**Results:**

A total of 9 studies (n=6, 67% published between 2021 and 2023) with quality rated as medium to high were included, of which 7 (78%) were randomized controlled trials (n=5, 71% were pilot and feasibility studies). All the included studies evaluated self-management interventions, most of them (n=7, 78%) specifically designed for older adults. The participants were aged between 65 and 75 years on average (mean 68.5, SD 3.5 y) and had been diagnosed with different types of CNCP (eg, osteoarthritis and chronic low back pain). Most of the included studies (5/9, 56%) involved the use of multiple eHealth modalities, with a higher use of web-based programs and video consulting. Only 1 (11%) of the 9 studies involved a virtual reality–based intervention. The evaluated interventions showed signs of effectiveness in the targeted biopsychosocial outcomes, and the participants’ engagement and ratings of satisfaction were generally positive. However, several research gaps were identified and discussed.

**Conclusions:**

Overall, of late, there has been a growing interest in the potential that eHealth multimodal interventions offer in terms of improving pain, physical, and psychosocial outcomes in older adults with CNCP. However, existing literature on this topic still seems scarce and highly heterogeneous, with few proper randomized controlled trials, precluding robust conclusions. Several gaps emerged in terms of the older population considered and the lack of evaluation of comorbidities.

**International Registered Report Identifier (IRRID):**

RR2-10.1101/2023.07.27.23293235

## Introduction

### Background

Chronic pain represents a substantial burden to health care systems and society [1]. According to recent estimates, its prevalence is especially high in older adults [2]. In fact, during recent decades, the prolongation of life expectancy has led to a substantial demographic revolution. According to global estimates [3], the number of people living beyond age 60 years will double to 2.1 billion by 2050 worldwide, and it is a trend that is bound to continue. Simultaneously, this process has entailed an increase in the prevalence of health conditions and symptoms usually associated with aging, such as chronic pain [2], which often results in significant physical disability, psychological disorders, cognitive decline, and sleep impairment [4,5]. Chronic pain negatively affects the quality of life of older adults [6], and, conversely, according to older people themselves, the absence of pain contributes to their psychological well-being [7]. Evidence suggests that between 25% and 50% of community-dwelling older adults and approximately 80% of older adults who are institutionalized experience pain, thus reflecting a contemporary clinical issue [8]. Unfortunately, there is a common disbelief, often shared by older adults themselves and even by health care professionals, that chronic pain is a natural and inevitable part of the aging process [9]. This can lead older adults to skip necessary care or to minimize or underestimate the presence of pain, with negative effects in terms of physical and mental health.

The assessment and treatment of pain in the older population are complex and challenging for health care providers because the pain typically occurs in the setting of physiological changes, multiple comorbidities, and polypharmacy, often limiting some therapy options, such as nonsteroidal anti-inflammatory drugs [10,11]. As a consequence, the focus of clinicians and researchers in recent years has been on a biopsychosocial model of chronic pain, according to which the experience of pain is determined by the complex interaction between biological, psychological, and social aspects [12-15]. The adoption of a multimodal approach is even recommended for likely a more effective management of persistent pain in older adults through combinations of both pharmacological and nonpharmacological therapies (such as psychological interventions, exercise therapy, and occupational therapy) [16-20]. To improve the accessibility to effective treatment programs for chronic pain, an important step forward has been made with the digital transformation that, in recent decades, has impacted various areas of society, including health care [21]. The term “eHealth” (or digital health) refers to “the use of information and communications technology in support of health and health-related fields” [22]. As for chronic pain treatment, different solutions have been developed and implemented with encouraging results, such as web-based programs, mobile health (mHealth), virtual reality (VR) systems, and video consulting [23,24].

The use of digital technologies in the field of chronic pain management among older adults is still little explored, especially regarding the potential effects of multimodal interventions (including both physical and psychosocial components) delivered by digital devices. In this regard, it should be noted that older adults, especially those aged ≥85 years, usually show concern and anxiety when using digital technologies due to many barriers to accessing and understanding these tools in light of the existing digital gap compared to the new generations [25-27]. Nevertheless, the adoption of digital technologies by older adults has significantly increased in the last 10 years; for example, the results of a recent survey in the United States showed that 96% of people aged 50 to 64 years and 75% of those aged ≥65 years were using the internet [28].

Given that the topic of eHealth for older adults with chronic pain has not yet been extensively reviewed and in light of the heterogeneous nature of the body of knowledge in this area, a scoping review of the literature is the most appropriate methodology to investigate this emerging field of research.

### Objectives

This scoping review aims to systematically map existing literature on eHealth multimodal interventions (including both physical and psychosocial components) designed for older adults with chronic noncancer pain (CNCP) to answer the following research questions (RQs):

- RQ1: What is the body of evidence (eg, in terms of the number and quality of studies)?
- RQ2: What are the gaps in current literature?
- RQ3: What kind of populations have been considered (eg, in terms of specific chronic pain characteristics and underlying conditions, age subgroups, and comorbidities)?
- RQ4: What are the main characteristics of the eHealth multimodal interventions (including physical and psychosocial components) used in older adults with CNCP?
- RQ5: What are the main outcomes and promising results of these interventions?

## Methods

### Overview

This scoping review was guided by the JBI methodology [29,30] and adhered to the PRISMA-ScR (Preferred Reporting Items for Systematic Reviews and Meta-Analyses extension for Scoping Reviews) guidelines and checklist [31]. The corresponding protocol has been posted on the medRxiv preprint platform [32].

### Search Strategy and Screening Procedure

Four electronic databases were searched for relevant articles from their inception up to August 2023: PubMed, Cochrane Central Register of Controlled Trials, Web of Science, and PsycINFO.

A combination of key terms related to the following four main topics was used as a search strategy: (1) eHealth (eg, telemedicine, telehealth, and mHealth), (2) psychosocial and physical (eg, psychotherapy, psychoeducation, physiotherapy, and physical activity) or multimodal (eg, multicomponent or multifactorial and mind-body therapy) interventions, (3) older people, and (4) chronic pain. The full search strategies used for the databases are presented in Multimedia Appendix 1.

An additional manual search of the references was carried out by screening reviews identified through the search strategy. Furthermore, forward citation tracking on the included articles via Google Scholar was performed on November 9, 2023, to broaden the findings and update the search.

All identified articles were exported to Rayyan (Rayyan Systems Inc), a web-based application designed to facilitate the systematic review process [33], and duplicates were removed. Two reviewers (ADL and SP) independently assessed titles and abstracts for eligibility. Full texts of the potentially eligible articles were retrieved and assessed against eligibility criteria by the same 2 independent reviewers. Doubts were discussed, and, where necessary, a third reviewer (VD or CP) was involved.

### Inclusion and Exclusion Criteria

Eligibility criteria were defined according to the population, concept, context framework [29] (Textbox 1). As for the age criterion, although some conventional “old age thresholds” exist (eg, 65 years old) [34-36], different age limits or specific age ranges have been used in the literature [37]. Thus, in this scoping review, we included all studies targeting specifically the older population without setting an age limit. However, as this is a scoping review, studies were included even when they had a sample with a mean age of ≥65 years (without any upper limit) of either sex.

**Eligibility criteria based on the population, concept, context framework.**

**Population**

1. Studies aimed at the older population, or the population included in the study had a mean age of ≥65 years, of either sex
2. Presence of chronic noncancer pain, with no limitations in terms of underlying clinical conditions

**Concept**

1. Multimodal interventions, involving both physical (eg, therapeutic exercise and functional training) and psychosocial (any intervention targeting ≥1 emotional, cognitive, behavioral, or interpersonal aspects) components, delivered (even partially) through any type of eHealth tool and targeting the following main outcomes: pain (eg, severity), emotional functioning (eg, emotional distress, anxiety and depressive symptoms, catastrophizing, kinesiophobia, and perceived self-efficacy), physical functioning (eg, functional mobility and endurance), and integrated outcomes (eg, health-related quality of life, well-being, general functioning, and disability)

**Context**

1. Any care setting (eg, primary care, outpatient, community, secondary care, or tertiary care)

Despite the lack of a shared definition in the literature, the umbrella term “multimodal” in the context of chronic pain management generally refers to “the combination of multiple therapeutic components, not necessarily provided by different operators” [17]. Specifically, in the context of this review, in accordance with the recent NICE (National Institute for Health and Care Excellence) guideline for chronic pain [19], we included eHealth multimodal interventions that have ≥2 components, including at least 1 physical (nonpharmacological) component and 1 psychosocial component.

Studies were excluded if they (1) were not aimed at the older population or, in the case of a study not explicitly aimed at the older population, the population included in the study had a mean age of <65 years; (2) focused on acute or cancer-related pain; (3) described a single-component intervention (ie, only physical or only psychosocial); and (4) did not use an eHealth modality to deliver (even partially) the intervention.

Journal articles were included if they were written in English or Italian. No limitations with regard to the year of publication were imposed. Both qualitative and quantitative studies were considered eligible for this review. The following types of studies were excluded: systematic reviews, narrative reviews, meta-analyses, bibliometric analyses, letters, case studies, books or book chapters, comments, editorials, congress abstracts or symposia proceedings, poster presentations, and dissertations.

In the cases of different papers presenting the same intervention by the same research groups, the article describing the main results of the eHealth intervention (often the more recent one) was included in the review and considered a reference paper in the Results section, while the previous or secondary papers (referred to as *secondary papers*) were excluded in the flowchart and whenever we reported quantitative information on the number of papers in the text. However, when useful data were reported in these secondary papers, contents were added to the text and in the corresponding tables.

### Data Extraction and Synthesis

Data were extracted from the selected articles by 2 independent reviewers (ADL and SP) using a data-charting table in Microsoft Excel that was approved by the research team. Any disagreement was resolved through discussion until a consensus was reached.

The extracted data included the following items: study characteristics (ie, authors, year of publication, and study design); information related to the sampled population (ie, age, sex, type of chronic pain, pain intensity and duration, main comorbidities, and setting); the intervention (ie, its conceptual basis, structure, main components, duration and format, specifying whether it is guided or unguided, and the providers involved); the type of eHealth tool used to deliver the intervention; the targeted outcomes (including older people’s experiences and perceptions of eHealth interventions, when applicable), the measurement tools, and the main results; the follow-up duration, when applicable; and, when involved, the type of control group. The extracted information was presented in tabular form along with a narrative summary that is in line with the scoping review’s objective.

### Assessment of Methodological Quality

Two independent reviewers (ADL and IP) evaluated all eligible studies using the Quality Assessment Tool for Studies with Diverse Designs (QATSDD) [38]. Any potential disagreement was discussed, with a third rater (VD or CP) adjudicating. The tool has shown good reliability and validity [38], and it allows the quality of research papers to be rated on a scale ranging from 0=*not at all* to 3=*complete* across 16 criteria. These criteria apply to studies that adopt different methodologies (ie, qualitative, quantitative, and mixed methods). For qualitative or quantitative research, the highest score is 42; and for mixed methods studies, the maximum score is 48. For each included article, the score attributed to each indicator and the articles’ overall quality score (ie, resulting from the sum of individual scores for each item) were provided. Moreover, to identify the items with higher and lower values, each item’s mean and SD were calculated in addition to the average quality score for all studies.

## Results

### Study Selection

**Figure 1:**
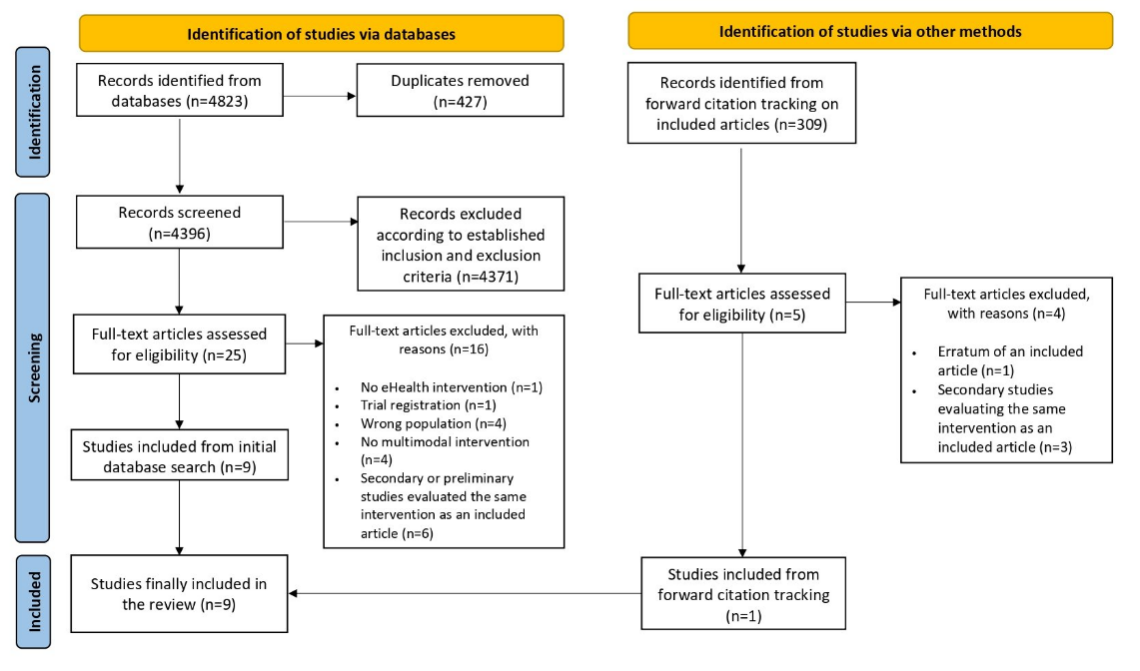
PRISMA (Preferred Reporting Items for Systematic Reviews and Meta-Analyses) 2020 flow diagram showing the identification and selection of studies.

The electronic literature search yielded 4823 records, from which 427 (8.85%) duplicates were removed. The remaining 4396 records were screened by title and abstract, and 4371 (99.46%) were excluded based on the inclusion and exclusion criteria. Finally, 25 records were selected for the full-text analysis, of which 16 (64%) were excluded for various reasons (refer to Figure 1 for details, and refer to Multimedia Appendix 2 [39-58] for the list of the full texts excluded and the reasons for exclusion). Thus, 9 articles from the initial database search were included. Of the 16 excluded articles, 6 (38%) were excluded because they focused on the same eHealth intervention described in a previous or secondary paper by the same research group. In addition, 309 papers were identified from the forward citation tracking methodology of included articles and then screened by title and abstract according to the inclusion and exclusion criteria. Of the 5 papers selected for the full-text analysis, 4 (80%) were excluded. Finally, 9 articles were included in this scoping review [59-67] (a preliminary study was excluded from the 9 articles identified from the initial database search and replaced with the study identified from the forward citation tracking).

### Characteristics of the Included Studies

Of the 9 included studies, 6 (67%) were published between 2021 and 2023 [59,62-66], whereas 1 (11%) was published in 2009 [61], 1 (11%) in 2015 [60], and 1 (11%) in 2016 [67]. Of the 9 studies, 4 (44%) were carried out in the United States [61-64]; 2 (22%) were conducted in Europe, specifically in Germany [65] and the United Kingdom [67]; and 1 (11%) study each was conducted in Canada [66], Australia [59], and Thailand [60]. Of the 9 studies, 7 (78%) were randomized controlled trials (RCTs) [59-65], most of which (n=5, 71%) were pilot and feasibility studies [61-65], while 2 (22%) were mixed methods studies that reported both qualitative and quantitative data [66,67].

### Population Characteristics of the Included Studies

Population characteristics of the included studies are presented in Table 1. Of the 9 studies, 7 (78%) evaluated interventions designed and proposed specifically for older adults [60-65,67], while the remaining 2 (22%) described interventions targeting a population with a mean age of ≥65 years [59,66]. In all the included studies, the average age of the population was between 65 and 75 years (Table 1). Where provided, the percentage of female patients in the studies ranged between 54.8% and 92.5%. In most of the included studies (7/9, 78%), participants were recruited from community-based settings (eg, older adult centers, services providers, and religious congregations) [61]; private health insurers [59]; research databases; and, via word of mouth, community locations serving older adults [64]. Conversely, in 2 (22%) of the 9 studies, research participants were outpatients at a general hospital [62] and an orthopedic clinic [66].

**Table 1:** Population characteristics (sociodemographic and clinical) of the included studies (n=9).

Study	Sample size (n); age (y), mean (SD), sex (%)	Pain: type; intensity^a^ (*measure*^b^); duration	Excluded health conditions	Other concurrent health characteristics	Setting
Bennell et al [59]	414; CG^c^: 65.3 (8.7), F^d^=67.2; IG^e^1: 65.4 (8.2), F=54.1; IG2: 64.1 (8.1), F=50.9	Knee OA^f^; moderate (*numeric rating scale*); IG1: median 5.0 (IQR 2.0-10.0) y, IG2: median 5.0 (IQR 3.0-10.0) y, and CG: median 4.0 (IQR 2.0-10.0) y	Conditions where the dietary intervention necessitated medical monitoring (eg, BMI ≥41 kg/m^2^ and type 1 or type 2 diabetes requiring medication except metformin, warfarin use); stroke or cardiac event in the past 6 months; unstable heart condition; fluid intake restriction; inflammatory arthritis and knee surgery within the past 6 months	Overweight or obesity as well as cardiac and respiratory issues; bladder or prostate or urologic, psychological, gastrointestinal, type 2 diabetes, and osteoporosis conditions	Community
Saraboon et al [60]	80; IG: 67.3 (6.3), F=92.5; CG: 67.5 (7.3), F=92.5	Knee OA; mild to moderate (*Knee Severity Scale*); NR^g^	Cognitive deficits; history of hip or knee surgery or treatment plans for knee surgery; knee joint injections in the 3 months before enrollment; serious illnesses or conditions	Overweight; comorbid conditions (%): IG: 52 and CG: 55	Community
Berman et al [61]	78; 65.8 (SD NR), F=87.2	A variety of underlying conditions; moderate (*Brief Pain Inventory–Short Form*); NR	NR	NR	Community
Doorley et al [62]	20; IG: 68.9 (5.9), F=66.7; CG: 70.8 (8.4), F=63.6	Any type of musculoskeletal chronic pain; moderate (*numeric rating scale*); >5 y=30%, 6-10 y=25%, and ≥11 y=45%	Medical illness expected to worsen in the next 6 months; serious mental illness; inability to walk without assistance	Cognitive impairment	General hospital
Fanning et al [63]	44; 68.8 (7.9), F=75	Chronic multisite pain; moderate (*PROMIS*^h^ *3-item* *version*); NR	Unstable weight; >1 fall in the previous year; impaired vision or cognition; severe medical illness; orthopedic surgery or joint replacement in the previous 6 months or planned to have such a surgery in the coming 6 months	Obesity	Community
Janevic et al [64]	46; 72.1 (7.2), F=89	Chronic musculoskeletal pain; moderate *(PROMIS* *43-item* *version*); ≥3 mo	Serious acute illness or hospitalization in the last month or planned major surgery in the next 3 months; significant memory difficulties that interfered with daily activities and with participation in the program	Underserved community; comorbid conditions: mean 6.0 (SD 2.2)	Community
Stamm et al [65]	22; IG: 75 (5.8), F=72.7; CG: 75.5 (4.4), F=54.5	Chronic back pain; mild (*numeric rating scale*); IG: mean 15.8 (SD 12.7) y and CG: mean 26.4 (SD 16.6) y	Intervertebral disc surgery in medical history; immobile or mobility possible only with assistance; sensory and motor failure; spinal malignancies, spondylitis, or spondylodiscitis; severe vestibular restrictions affecting the ability to balance	NR	Community
Godziuk et al [66]	53; 65 (7), F=71.7	Knee OA; moderate to severe (*3-point scale*); >5 y=66.1%	BMI <25 kg/m^2^; prior joint arthroplasty	Overweight or obesity	Outpatients at orthopedic centers
Pearson et al [67]	83; 67 (SD NR), F=67	OA; NR; <1 y: 6%, 1-5 y: 33%, 5-10 y: 31%, >10 y: 28%, and unknown: 2%	NR	NR	Community

^a^Pain level reported by the authors of the included studies.

^b^In this column, the pain intensity measures used in each study are italicized.

^c^CG: control group.

^d^F: female.

^e^IG: intervention group.

^f^OA: osteoarthritis.

^g^NR: not reported.

^h^PROMIS: Patient-Reported Outcomes Measurement Information System.

With regard to the patients’ pain characteristics, the reported information was heterogeneous and varied significantly among the included studies; however, all participants experienced chronic pain with different levels of severity. Of the 9 studies, 4 (44%) included participants with knee osteoarthritis [59,60,66] or with osteoarthritis affecting various body sites (eg, hip and back) [67]. The remaining studies (5/9, 56%) recruited participants with different types of CNCP. More in detail, in the study by Berman et al [61], the most common causes of chronic pain reported by participants were arthritis, spinal stenosis or degenerative disc problems, previous injuries or surgery, and sciatica. Other types of pain included were chronic back pain [65], chronic musculoskeletal pain [62,64], and chronic pain in at least 1 of 3 areas (back, hip, or knees) [63].

### Main Characteristics of the Interventions in the Included Studies

The main characteristics of the eHealth interventions in the included studies are presented in Multimedia Appendix 3 [59-67]. Of the 9 interventions, 3 (33%; ie, Active Brains–Fitbit [62], Mobile Intervention to Reduce Pain and Improve Health II [MORPH-II] [63], and Positive Seniors Using Technology to Engage in Pain Self-Management [64]) were investigated and slightly adapted in various studies by the same research group. Herein, we describe the intervention presented in the main study (ie, the one included in the review), even reporting any relevant adjustment to the intervention made in secondary studies (ie, changes reported in italics in Multimedia Appendix 3).

Overall, all included studies assessed multimodal self-management interventions aimed at promoting more effective self-management of pain symptoms and improved health-related quality of life (HRQoL) through health behavior modification techniques and strategies. These programs are diverse in terms of theoretical frameworks, contents, specific outcomes, and targeted populations. Despite the differences, these interventions are generally structured in different modules, characterized by both psychosocial and physical components and activities, the content of which covers to a varying extent the following three areas:

1. Health behavior education about pain physiology, the effects of pain, and treatment options; the benefits of physical activity; nutrition; sleep hygiene; stress management; and healthy lifestyles counseling
2. Self-management cognitive behavioral skills, such as mindfulness practices, relaxation exercises, activity pacing, goal setting, and positive thinking
3. Physical activity programs and muscle-strengthening exercises [60,65-67] that often involve the adoption of wearable activity monitors (eg, Fitbit devices, accelerometers, and actigraph units) [59,62-64]

In addition to this content, some of the studies (3/9, 33%) also provided nutritional and weight loss programs, aimed at promoting a healthy and active lifestyle in older patients with chronic pain and overweight or obesity [60,61,63]. In some of the cases (2/9, 22%), video consulting with dietitians or nutritionists was offered as part of an individualized treatment plan [59,66]. Intervention duration varied across the studies, ranging from 4 weeks [65] to 6 months [59].

The interventions were administered individually in two-thirds of the studies (6/9, 67%) [59,61,64-67], in a group setting in 1 (11%) of the 9 studies [62], and through a mixed format in the remaining studies (2/9, 22%) [60,63]. More than two-thirds of the interventions (7/9, 78%) were delivered fully remotely [59,61-64,66,67]; the remaining interventions (2/9, 22%) adopted a blended modality [60,65].

### eHealth Modalities Adopted for the Interventions

Regarding the type of eHealth modalities, those used in the included studies to deliver the intervention have been grouped into 4 categories. Specifically, of the 9 studies, 5 (56%) used web-based modalities (ie, self-guided or therapist-assisted programs to improve knowledge and provide support, care, or treatment to a diverse population with a range of health problems by using websites or web applications [23,68]); 4 adopted mHealth modalities (ie, “medical and public health practice supported by mobile devices, such as mobile phones, patient monitoring devices, Personal Digital Assistants [PDAs], and other wireless devices” [69]); 5 (56%) used teleconsulting, such as telephone consulting (ie, “telephone support from health practitioners” [70]) and video consulting (ie, “the utilization of online internet networks to access real-time, high-quality video and audio connections” [71]); and 2 (22%) studies used “other devices,” including VR (ie, “a three-dimensional virtual world that may be explored, interacted with, and manipulated by the person” [72,73]; Table 2).

**Table 2:** eHealth modalities used to deliver the interventions in the included studies.

Study	Modality			
	Web	mHealth^a^	Video or teleconsulting	Other devices
Bennell et al [59]	Website	Wearable activity monitor (Fitbit)	Videoconferencing platform^b^	—^c^
Saraboon et al [60]	—	—	—	Video CDs
Berman et al [61]	Website	—	—	—
Doorley et al [62]	—	Wearable activity monitor (Fitbit; ActiGraph)	Videoconferencing platform^b^	—
Fanning et al [63]	—	Tablet and smartphone app; wearable activity monitor (Fitbit); wireless weight scale	Videoconferencing platform^b^	—
Janevic et al [64]	Web-based videos^b^ (website)	Wearable activity monitor	Telephone calls	—
Stamm et al [65]	—	—	—	VR^d^ system
Godziuk et al [66]	Emails^b^; web platform	—	Webinar; telephone and video consulting	—
Pearson et al [67]	Website	—	—	—

^a^mHealth: mobile health.

^b^Main eHealth modality.

^c^Not present.

^d^VR: virtual reality.

These eHealth modalities were not mutually exclusive, and most of the studies (5/9, 56%) used a combination of some of these modalities to deliver the intervention (such as a combination of web-based modality and video consulting; mHealth and video consulting; or web-based modality, video consulting, and wearable activity monitor) [59,62-64,66]. In some cases (2/9, 22%), the intervention’s core contents (eg, videos and booklets) were sent directly to participants via email, providing additional resources delivered through web platforms, video consulting [66], or telephone sessions [64]. In other cases (3/9, 33%), the main components of the program were administered during web-based sessions through the use of videoconferencing platforms, including the support of supplemental digital tools, such as wearable activity trackers, websites, and smartphone apps [59,62,63]. Of the 4 studies that used only 1 modality, in 2 (50%), participants were asked to access a website to complete the intervention’s modules [61,67]; in 1 (25%), the treatment was provided through a VR system in a research setting [65]; and, finally, in 1 (25%) study, the use of video CDs was planned as part of an intervention program delivered during in-person group meetings [60].

### Psychosocial, Physical, and Integrated Outcomes Targeted by the eHealth Interventions

Multimedia Appendix 4 [59-67] presents the outcomes considered in the included studies, which were classified into 5 categories: *pain*, *psychological*, *physical*, *integrated* (including perceived biopsychosocial outcomes), and *other outcomes*. For each study, the outcome measures used to assess each variable and the related main significant or not significant results are also provided.

Among the pain outcomes, pain intensity was investigated in all studies. The targeted psychological outcomes were quite heterogeneous among the studies, but, overall, they consisted of emotional distress (eg, anxiety and depressive symptoms) and pain-related psychological variables. As for the former, 3 (33%) of the 9 studies assessed anxiety and depressive symptoms [59,61,62]. The pain-related psychological outcomes considered in the studies were perceived self-efficacy [61,62,64,66], pain catastrophizing [62], pain acceptance [62], kinesiophobia [62,65], illness representation [60], and self-awareness of responses to pain [61]. Other psychological outcomes assessed were cognitive functioning, mindfulness and self-compassion skills [62], coping, resilience [64], and self-determinative (autonomy, competence, and relatedness) needs satisfaction and frustration [63]. The evaluated physical outcomes were physical function and motor skills [59,60,62-64]. In the studies involving participants who were obese or overweight, weight was also monitored [59,60,63]. Among the integrated outcomes, perceived health status and HRQoL [65,66], pain interference [61,63,64], functional capacities [62,65], well-being [66], social participation [64] and social functioning [62], and health behavior related to pain [60] were included. Other variables concerned satisfaction with, and usefulness of, the intervention; acceptability and use of the eHealth tool; engagement; and participation [62-67].

### Signs of Effectiveness of the eHealth Interventions

Among the included articles, the RCTs (7/9, 78%) compared the eHealth interventions with other control conditions such as information only, waiting list, conventional therapy, and so on [59-65] (Multimedia Appendix 4). Only the study by Bennell et al [59] included a long-term follow-up assessment. The results are described herein in the following order: first, the RCTs (2/9, 22%); next, the pilot or feasibility RCTs (5/9, 56%); and, finally, the mixed methods studies (2/9, 22%).

In the study by Bennell et al [59], looking at the between-group mean difference, both the exercise and diet+exercise programs revealed greater increases in pain intensity reduction (diet+exercise: *P*<.001; exercise: *P*=.01) and physical function (diet+exercise: *P*<.001; exercise: *P*<.001) compared with an information control group at 6 months after treatment; the diet+exercise program was also superior to the exercise-only program for these outcomes (*P*=.005). Up to 79.4% of the participants in the diet+exercise group and 58.2% in the information control group achieved the minimal clinically important difference (MCID) in overall knee pain (1.8 units on the numeric rating scale [NRS]; *P*<.001); and 74.6% of the participants in the diet+exercise program, 66.2% in the exercise program, and 34.5% in the control group achieved the MCID in physical function (6 units on the Western Ontario and McMaster Universities Osteoarthritis Index; *P*<.001). Similar results were found at 12-month follow-up; in addition, 78.2% of the participants in the diet+exercise group and 66% in the exercise group achieved the MCID on the NRS (*P*=.02), while 78.1% of the participants in the diet+exercise group and 63.8% in the exercise group achieved the MCID on the Western Ontario and McMaster Universities Osteoarthritis Index (*P*=.045).

In the study by Saraboon et al [60], the multifactorial intervention program showed a statistically significant increase in osteoarthritis knowledge, illness representation, health behavior, movement ability, and joint range of motion, as well as a reduction in knee pain and body weight (*P*<.001). All these variables were found to have significant differences between the intervention and control (information only) groups (*P*<.001).

Looking at the pilot or feasibility RCTs [61-65], Berman et al [61] found statistically significant improvements in pain intensity (worst pain: *P*=.01; least pain: *P*=.05 for the intervention group and *P*=.01 for the control group; average pain: *P*=.01 for the intervention group and *P*=.05 for the control group; average intensity: *P*=.01 for the intervention group and *P*<.001 for the control group) and pain interference (*P*=.01) in both the web-based intervention and waiting list control groups as well as increases in “confidence with using nonmedical self-care techniques to manage pain” (*P*<.001) in the intervention group.

In the study by Doorley et al [62], preliminary signs of improvement for the Active Brains–Fitbit web-based intervention group were found in pain intensity (pain intensity with activity: *P*=.05; Cohen *d*=1.0) as well as cognitive (*P*=.05; Cohen *d*=0.8) and physical (average steps: *P*=.04; Cohen *d*=0.4; physical activity intensity: *P*=.04; Cohen *d*=1.2) functioning. Conversely, the health enhancement program control group showed a significant decrease in physical functioning (average steps: *P*=.02; Cohen *d*=0.3).

Consistent with promising results from a series of earlier trials [52,54], Fanning et al [63] observed a large effect on competence satisfaction (*P*<.01; η^2^=0.22), average daily steps (*P*=.02; η^2^=0.23), and postural shifts (*P*=.02; η^2^=0.24) in the intervention group but not in the control group.

Janevic et al [64] found greater significant improvements in the web-based intervention group versus the waiting list control group in measures of pain self-efficacy (*P*=.007; η_p_^2^=0.155) and pain interference (*P*<.001; η_p_^2^=0.166). Moreover, significantly more participants in the intervention group compared with participants in the control group reported “better” or “much better” global functioning (*P*<.001) and pain (*P*=.003) after treatment. The minimally important difference of 2.5 points on the PROMIS (Patient-Reported Outcomes Measurement Information System) pain interference subscale was achieved by 53% of the intervention group participants and 17% of the control group participants (*P*=.02).

In the study by Stamm et al [65], only the VR intervention was associated with a significant increase in functional capacities (*P*=.03; *r*=0.67), and only the conventional control condition showed a significant increase in general mental health (*P*=.01; *r*=0.81). Although not statistically significant, both intervention and control groups showed a reduction in pain intensity after treatment (*P*=.50 and *P*=.07, respectively). However, considering that, on average, a reduction of approximately 30% on the NRS represents a clinically important difference for chronic low back pain, the intervention group showed a reduction in pain intensity of only 18.02% on the NRS, whereas the control group achieved a reduction of 43.64%.

Finally, although the study by Godziuk et al [66] presents specific methodological limitations (ie, it is a single-arm study), the multimodal intervention in this study was associated with an increase in perceived self-efficacy for managing symptoms (*P*=.003) and daily activities (*P*<.001), HRQoL components of pain (*P*=.02) and physical functioning (*P*<.001), and an understanding of arthritis symptoms (*P*<.001), as well as with a decrease in interest in total knee arthroplasty (*P*<.001).

### Feasibility and Acceptability of the eHealth Interventions

Feasibility, acceptability, or participants’ satisfaction with the intervention was evaluated in 8 (89%) of the 9 included studies [59,61-67] (Multimedia Appendix 4).

In the study by Bennell et al [59], participants in the exercise group and those in the diet+exercise group (92.7% and 97%, respectively) were more satisfied with their care compared to the participants in the control group (16.7%) at 6 months after treatment (*P*<.001), with similar results even at 12 months.

In the study by Berman et al [61], most of the participants found the web-based intervention to be highly useful (81.4%) and user-friendly (88.4%).

In the study by Doorley et al [62], the Active Brains–Fitbit group met the criteria for “excellent” on all benchmarks set a priori by the authors (eg, feasibility of recruitment, program acceptability, and credibility), except for treatment expectancy, and was rated both feasible and satisfactory in the qualitative results.

In the study by Fanning et al [63], both qualitative and quantitative data supported the feasibility and acceptability of the MORPH-II program. More in detail, support for the feasibility of the program included a high attendance rate for the remote sessions (82.5% on average) and a high retention rate for follow-up testing (90.9%). Technological aspects of the MORPH-II program were evaluated as acceptable and usable (with an average score on the System Usability Scale of 77, which is categorized as “good to excellent,” and a median score of 85, which is classified as “best imaginable” usability). Qualitative interviews revealed overall positive feedback on the program, which was described as “beneficial” and “life changing.”

In the study by Janevic et al [64], participants provided overall positive feedback about the Positive Seniors Using Technology to Engage in Pain Self-Management intervention, with 95% of the participants strongly agreeing or agreeing that they increased their understanding of pain management and that the program helped them reach their pain management goals. Moreover, the retention rate was 90%, while, in terms of engagement, the results showed a mean of 5.7 completed sessions (out of 7), with 95% of the participants reported to have watched all program videos.

In the study by Stamm et al [65], users of the VR system experienced a “higher degree of immersion” (average score of 19.09 points on the Technology Usage Inventory) and rated the VR program as at least above average on most of the evaluation criteria (eg, attractiveness, perspicuity, efficiency, dependability, and stimulation) and as mostly good and excellent in attractiveness and perspicuity.

In the study by Godziuk et al [66], the intervention’s resources—especially email content—were positively perceived as appropriate and tailored for patients with knee osteoarthritis. However, concerns related to privacy and entering personal information on web platforms emerged. Finally, the mixed methods study by Pearson et al [67] aimed at co-designing a web-based version of a multimodal intervention for chronic pain relying on qualitative data from surveys, focus groups, and semistructured interviews. Briefly, according to these data, older people seemed more likely to accept a web-based program as a supplement to, rather than as a replacement for, a face-to-face intervention.

### Assessment of the Methodological Quality of the Included Studies

Overall, the QATSDD score ranged between 71.4% (mean raw score=30) [60] and 92.9% (mean raw score=39) [62] (Multimedia Appendix 5 [59-67]). The average quality score for all studies was 82.1% (raw score=34.5). Variations in quality among the studies mainly concerned the following items: evidence of sample size considered in terms of analysis, the presence of a statistical assessment of the reliability and validity of measurement tools, and a critical discussion of the strengths and limitations. The lowest QATSDD single-item score related to user involvement in the study design, which was considered only in 5 (56%) of the 9 studies (item score: mean 1.4, SD 1.4) [62-65,67]. In addition, the statistical assessment of the reliability and validity of measurement tools was limited in most of the studies (6/9, 67%; item score: mean 1.6, SD 1.1).

## Discussion

### Principal Findings

This scoping review systematically mapped existing literature regarding eHealth multimodal interventions (including both physical and psychosocial components) in older adults with CNCP. The population involved, potential gaps in the literature, and the main interventions’ characteristics, as well as the results in terms of the signs of effectiveness, feasibility, and acceptability, were explored and described. Nine studies, mainly of recent publication (6/9, 67% were published between 2021 and 2023), were included in this scoping review. Although the implementation of digital solutions is increasingly occurring in several health care settings [74], the use of such technologies specifically targeting multimodal interventions for CNCP management in the older population still seems to be relatively rare and very recent.

The dearth of literature on eHealth solutions for CNCP dedicated to the older population might be explained by several factors. First, the negative stereotypes that portray older adults as a homogeneous group mostly characterized by vulnerability, inactivity, and cognitive decline may deter their inclusion in research, especially when it concerns digital technologies [75]. Second, the fact that the older population is overall less digitally literate than younger cohorts [76] might have discouraged researchers and investors from financing the research and development of digital health solutions specifically targeted at older people [77]. It is also noteworthy that, in the last decade, we have been facing a technological revolution with the rapid growth of digital health solutions which, on the one hand, brings major benefits and positive changes for society and the health care system but, on the other hand, poses a great challenge in terms of economic, ethical, and regulatory issues [78]. In particular, the recent development of therapeutic options being classified as a medical device (MD; ie, “any instrument, apparatus, appliance, software, implant, reagent, material or other article intended by the manufacturer to be used, alone or in combination, for human beings for one or more...specific medical purposes” [79]) or a digital therapeutic (DT; ie, “health software intended to treat or alleviate a disease, disorder, condition, or injury by generating and delivering a medical intervention that has a demonstrable positive therapeutic impact on a patient’s health” [80]) has been forcing countries to adapt their regulatory systems in terms of definitions, terminology, requested evidence, and payment and reimbursement rules, with tremendous differences among countries [81,82]. Although some solutions have been recently introduced for different pain conditions [83,84], no MD or DT specifically developed for the management of chronic pain in the older population exists, to the best of our knowledge. Despite this, although not specific for the target population of this scoping review, such a complex scenario, together with the lengthy development process for these new devices, may have impacted the ease of conducting studies in this field, especially when added to the unique challenges linked to the older population (eg, negative stereotypes and digital literacy).

The quality of the studies included in this scoping review, in general, was rated as medium to high according to the QATSDD tool. The main strengths were the clear description of the research aims or objectives, setting, procedure for data collection, recruitment data, and the fit between the stated RQ and the methods of data collection and analysis selected. The main limitation of the studies was the very limited statistical assessment of the reliability and validity of the measurement tools. Moreover, the user involvement in the research or intervention design was somewhat considered only in just over half of the included studies (5/9, 55%). Specifically, in those studies meeting this criterion, qualitative methods (eg, surveys, focus groups, and semistructured interviews) were used as a first step to gain a better understanding of older adults’ preferences and needs and to consequently develop tailored programs. This is in line with the relevance of using participatory approaches when developing interventions for chronicity [85] and with the recommendation to adopt a person-centered approach by including end users in the design process of the intervention from the early stages to improve feasibility, acceptability, and satisfaction with the innovative digital technology [86].

A few issues regarding the characteristics of the populations covered by the studies should be taken into account. First, although our review was intentionally broad in considering the older population, with no age limits established, it is interesting to note that the average age of the participants included in the studies was between 65 and 75 years [87]. Therefore, other subgroups, including those aged 75 to 84 years and especially those aged ≥85 years, seem poorly represented in this field, suggesting an area of further research. Indeed, such cohorts may vary deeply in terms of physical and psychosocial (eg, needs, goals, and expectations) characteristics [88] as well as age-related differences with respect to the determinants of psychological well-being [89]; consequently, this potential uniqueness needs to be taken into account when designing tailored interventions. This becomes even more important when considering that the number of digitally literate people aged ≥75 years is expected to significantly increase in the future as the world’s population ages, and technology continues to advance. Therefore, it would be worthwhile to further focus on the diversity within the older population in this field, broadening the scope of interest of research dedicated to the older population to include people aged ≥75 years, who are more likely to be excluded from the evaluation of new technologies intended to meet the needs of the older population [7,90].

Moreover, in the included studies targeting the older population, the lower age limit varied substantially. Indeed, although the eHealth intervention was specifically designed for older adults, some of the studies considered populations with a minimum age of 50 years [60,67] or 55 years [61,63]. This is coherent with the lack of an established chronological age to define the older population and the high variability in the lower age limit, even after accounting for the differences among countries [37]; however, this poses a challenge for comparability among studies and underscores the need for studies on the older population to provide a more detailed description of results based on age distribution.

Second, the samples were composed primarily of female individuals, although this is consistent with CNCP epidemiology [91]. Third, the included studies were highly heterogeneous with respect to the types of CNCP included and also when considering the comorbid conditions. As regards the distribution of underlying conditions, 44% (4/9) of studies targeted participants with a specific diagnosis of osteoarthritis (ie, mainly of the knee and to a lesser extent of other body sites); in the remaining studies (5/9, 56%), different types of CNCP (eg, chronic musculoskeletal pain and chronic low back pain) were considered. On average, participants with mild to moderate levels of pain were recruited, except for a study in which approximately 70% of the participants self-identified their pain as severe [66]. All studies necessarily used self-reported methods to evaluate pain, pain being a subjective experience. Although it is necessary to respect patient pain reports, it has to be considered that complex factors influence how people report and interpret numerical pain ratings (eg, due to the “peak-end phenomenon” [92]), thus potentially introducing bias into the pain assessment, which should be accurately considered when interpreting the results [93,94].

With regard to comorbidities, it is particularly interesting to note that the chronic pain and cognitive decline comorbidity was addressed by only 1 (11%) of the 9 included studies [62]. This is surprising, considering that cognitive impairment has been found to be significantly and bidirectionally associated with CNCP in older adults [95] and that both of these health conditions show a high prevalence among this age group [95]. Moreover, considering that the world’s population continues to age, it can be assumed that an increasing number of people will experience such conditions in the future [95]. As for the study by Doorley et al [62], the authors developed and adapted a mind-body activity program to the unique needs of older people with both chronic pain and cognitive impairment; for example, they introduced cognitive functioning skills aimed at developing compensatory strategies for cognitive deficits and promoting intellectual stimulation. Overall, the aforementioned factors, as well as the large heterogeneity of research methodologies among the included studies, make it challenging to generalize the findings to the entire older population with CNCP. Finally, it is noteworthy to observe that none of the included studies carried out any assessment of frailty, although this state of vulnerability increases steadily with aging, and it is significantly associated with pain conditions [96].

To conclude, the aforementioned factors (eg, common comorbidities, the presence of frailty, and possible needs related to decreased digital literacy) should be considered when tailoring interventions for the older population to encourage the participation of older people with different characteristics.

**Figure 2:**
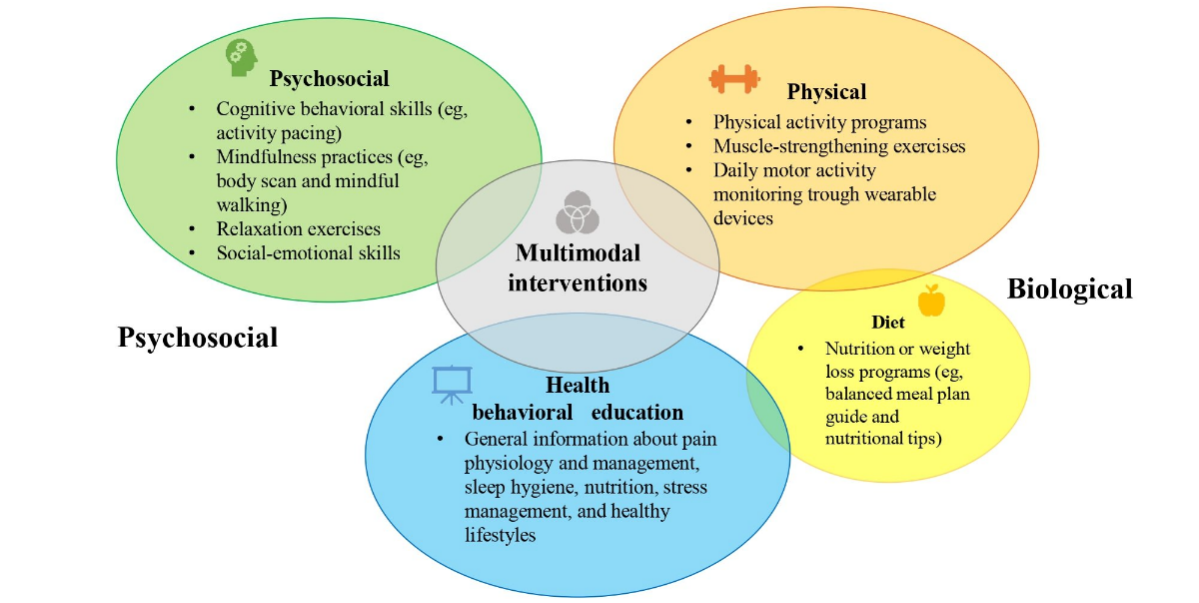
Graphical illustration of the main biopsychosocial components and strategies that emerged in the included eHealth interventions.

Regarding the evaluated eHealth interventions, these programs differ in terms of theoretical basis, contents, specific outcomes, and targeted populations, but they are inspired by a common biopsychosocial approach consistent with the current multidimensional conception of CNCP management [17,19,97]. This common framework finds expression through the implementation of both physical and psychological activities, broadly aimed at supporting more effective chronic pain self-management skills and ultimately at promoting an increased well-being and a better quality of life. In Figure 2, we present a graphical representation to illustrate the biopsychosocial components and strategies considered in the eHealth multimodal interventions included in this scoping review. The recent and increasingly widespread integrated pain team models promote the adoption of both pharmacological and nonpharmacological treatment options within patient-centered care settings [98], highlighting the importance of the provider-patient relationship, where the patients should be encouraged and empowered to effectively manage their health care. Regarding this, in 5 (56%) of the 9 studies included in this scoping review, a training and orientation phase on digital health tools was provided [61-64,66]. Moreover, in 7 (78%) of the 9 studies, at least 1 health care professional was available to offer video consultations, supervision, prompts, and support for the entire duration of the intervention, especially with respect to any technology-related questions. These aspects are in line with recent evidence, according to which older adults’ engagement with eHealth interventions might be optimized by providing social support to overcome potential technical barriers [99]; thus, considering these aspects might improve the practical application of eHealth solutions in the clinical context. Another factor that seems to contribute to older people’s commitment to a digital health intervention is the support from family members or caregivers, especially in assisting them during their initial exposure to these technologies [99]. However, only 1 (11%) of the 9 included studies considered the active involvement of relatives in supporting older adults to navigate technology-related challenges [62].

As for the interventions’ delivery formats, they ranged from fully remote programs (ie, completely based on an eHealth modality) to programs in which the digital health tool was a component of broader in-person treatment (eg, the use of VR or video CDs as part of in-person meetings). More in detail, the most common eHealth modality types used for the interventions were web-based programs (eg, websites and web platforms) and video consulting, followed by VR, which was applied in only 1 (11%) of the 9 studies. With respect to this last point, it should be noted that there is a lack of literature investigating VR-based multimodal interventions in the specific context of chronic pain and especially in the older population [100]. However, most of the included studies (5/9, 56%) involved multiple modalities (eg, by providing additional digital devices, such as wearable activity monitors, or smartphone apps). Some recent evidence suggests that older adults may be more prone to engage in eHealth interventions as long as these modalities do not completely replace face-to-face usual care [99]. Considering these data, video consulting may be a particularly acceptable digital health alternative for older people to be used in clinical practice because it allows for direct and synchronous contact with health care providers. Although the studies included in this scoping review did not consider these aspects, it would be interesting to consider the attitudes of health care professionals with respect to integrating some of these digital tools with usual treatments in clinical practice. In this regard, there is evidence of how the negative and ageist attitudes of health care providers may influence how the older adults are perceived in relation to the use of digital tools and consequently impact the actual adoption of these tools in clinical practice [75].

Overall, the evaluated interventions showed signs of effectiveness in the targeted pain, psychological, physical, and integrated outcomes. However, on the one hand, the limits of the methodological designs of the studies and, on the other hand, the heterogeneity of the outcomes, measures used to evaluate the outcomes, populations, and interventions considered in the studies hinder us from drawing conclusive results. In particular, with regard to the design of the 9 studies, it should be noted that only 2 (22%) were proper RCTs, whereas 5 (56%) were pilot and feasibility RCTs. In particular, the results of the 2 RCTs, both regarding chronic knee osteoarthritis pain (with overweight or obesity), suggested that multimodal education and exercise programs lead to improvement in diverse outcomes, including pain [59,60]. However, in the study by Bennell et al [59], the eHealth programs were completely delivered in a telehealth format, while Saraboon et al [60] only partially introduced the use of remote digital tools.

In line with these results, preliminary signs of effectiveness emerged from the RCTs; for example, summarizing the results for pain outcomes, a web-based intervention demonstrated positive effects on at least 1 pain measure in the studies by Doorley et al [62] and Janevic et al [64]; in addition, an improvement in confidence in managing pain was observed in the study by Berman et al [61]. In the study by Fanning et al [63], although there was no improvement in pain, the MORPH-II intervention led to increased perceived competence and physical activity. However, due to the limits intrinsic to their nature, these pilot and feasibility studies do not provide conclusive evidence on the effectiveness of the provided eHealth interventions, but they might serve as a guide in the implementation of future full-scale effectiveness studies (as mentioned in 3/9, 33% of the included studies [62-64]).

By contrast, these studies, together with the mixed methods studies, might be considered useful to examine the feasibility of the studied approach. As regards the feasibility of the interventions, when evaluated, the participants’ engagement, satisfaction, and feedback were generally positive, thus supporting the potential feasibility and acceptability of eHealth multimodal programs in older adults with CNCP.

Finally, only 3 (33%) of the 9 studies [59,64,65] considered the clinical relevance of the interventions, using diverse cutoffs for the MCID in pain (and in n=1, 33%, even function), and in 2 (67%) of these 3 studies, suggestions for improvement emerged [59,64]. In general, although the evaluation of clinical relevance is central for clinical guideline development and the interpretation of results, the introduction of the MCID in chronic pain research seems still underused, and more attention should be given to its appropriate methodological use [101]. As regards the use of VR for older adults with chronic back pain, it is interesting to note that only the control group achieved clinical relevance on this score in the study by Stamm et al [65], with interesting insights provided by the authors on the potential limits of this eHealth tool, which should be better considered to improve the effective application of VR in this context.

To sum up, not only are studies targeting eHealth interventions for chronic pain among the older population scarce, but the number of confirmatory RCTs is also especially limited, suggesting gaps to be covered in future research.

### Strengths and Limitations

This scoping review followed a systematic and structured theoretical framework to map the existing evidence regarding eHealth multimodal interventions for CNCP in older adults, in line with the JBI methodology [29,30]. In addition, it was reported in line with the PRISMA-ScR guidelines [31]. The review highlighted several gaps in this research field, suggesting areas that require more in-depth attention with regard to the implementation of eHealth multimodal interventions for CNCP in older adults. Finally, it provided a systematic synthesis of findings from both technical and clinical perspectives through the collaboration of a multidisciplinary team of researchers and clinicians.

However, this review has some limitations. First, because gray literature was not searched, any unpublished or ongoing studies are likely to have been missed. Although, to the best of our knowledge, MDs or DTs targeting chronic pain in the older population do not exist, other eHealth tools may have been developed by companies and investors but not tested in published studies and therefore not found within scientific articles. Second, only articles published in English or Italian were selected. In this regard, it should be noted that there is some evidence showing that excluding non-English literature from evidence syntheses does not alter conclusions [102]. Third, the adoption of selective criteria, such as the focus on the older population (refer to the Principal Findings section) and on multimodal interventions with regard to the inclusion of the studies, may have contributed to the paucity of studies identified. Regarding this last aspect, considering the relevance of adopting a multimodal approach to achieve optimal results in CNCP management, our aim was to explore the combined use of physical and psychosocial components through eHealth interventions. However, it is important to note that physical and psychosocial interventions may be proposed as stand-alone interventions and that our review did not include studies discussing only physical or only psychosocial interventions, which might be considered in a future exploration of the literature, even discussing how they might be integrated with each other and comparing the benefits of a single-component intervention versus a multimodal intervention.

### Conclusions

To conclude, growing interest has been shown in the potential that eHealth multimodal interventions offer in terms of improving CNCP management in older adults, although there is a dearth of studies in this field. The studies included in this scoping review mainly involved people aged 65 to 75 years, while those aged ≥75 years seem to be vastly underrepresented in this field. Similarly, only a few studies (6/9, 67%) considered the presence of comorbidities, particularly cognitive decline, and no study conducted an assessment of frailty. Moreover, existing literature seems to be limited by the small number of RCTs evaluating eHealth interventions for chronic pain among the older population and highly heterogeneous in terms of study designs, contents, measurement instruments, outcomes, and targeted populations, heavily limiting the possibility to draw robust conclusions. Nonetheless, our review indicates the potential of eHealth multimodal interventions in improving several pain-related outcomes in the older population. As this is an emerging and evolving field of research, several gaps and unresolved research issues remain that need to be fully and deeply addressed by future research.

## Appendix

Multimedia Appendix 1 Search strategy.

Multimedia Appendix 2 Full texts excluded and reasons for exclusion.

Multimedia Appendix 3 Main characteristics of the included eHealth interventions.

Multimedia Appendix 4 Targeted outcomes, measures, and main results of the included studies.

Multimedia Appendix 5 Assessment of methodological quality.

Multimedia Appendix 6 PRISMA-ScR-Checklist.

## Abbreviations

CNCP: chronic noncancer pain
DT: digital therapeutic
HRQoL: health-related quality of life
MCID: minimal clinically important difference
MD: medical device
mHealth: mobile health
MORPH-II: Mobile Intervention to Reduce Pain and Improve Health II
NICE: National Institute for Health and Care Excellence
NRS: numeric rating scale
PRISMA-ScR: Preferred Reporting Items for Systematic Reviews and Meta-Analyses extension for Scoping Reviews
PROMIS: Patient-Reported Outcomes Measurement Information System
QATSDD: Quality Assessment Tool for Studies with Diverse Designs
RCT: randomized controlled trial
RQ: research question
VR: virtual reality
