# Supplementary material for: eHealth-Integrated Psychosocial and Physical Interventions for Chronic Pain in Older Adults: Scoping Review
Source: J Med Internet Res. 2024 Jul 29;26:e55366. doi: 10.2196/55366 (PMC11319891; doi:10.2196/55366)
Supplement: Multimedia Appendix 1 [file jmir_v26i1e55366_app1.pdf]

Comprehensive search strategies

APA PsycInfo

1	telemedicine.mp. or exp Telemedicine/
2	(telehealth or tele-health).mp.
3	exp Mobile Health/ or m-health.mp.
4	e-health.mp. or exp Electronic Health Services/
5	ehealth.mp.
6	exp Internet/
7	exp Websites/ or web.mp.
8	online.mp. or exp Online Therapy/
9	app.mp.
10	exp Computers/ or computer.mp.
11	exp Technology/ or exp Virtual Reality/
12	exp Augmented Reality/
13	1 or 2 or 3 or 4 or 5 or 6 or 7 or 8 or 9 or 10 or 11 or 12
14	psychotherapy.mp. or exp Psychotherapy/
15	(psychological or psychosocial).mp.
16	(intervention* or therap* or treatment*).mp.
17	15 and 16
18	(relaxation and (technique* or therap*)).mp.
19	exp Relaxation Therapy/
20	exp Mindfulness-Based Interventions/ or exp Mindfulness/ or Mindfulness.mp.
21	(psychoeducation or "psycho-education").mp.
22	exp Psychoeducation/
23	exp Behavior Therapy/ or exp Cognitive Behavior Therapy/

24 ("cognitive behavioral therapy" or "cognitive-behavioral therapy" or "cognitive behavior therapy").mp.

25 (behavior and (intervention* or therap* or treatment*)).mp.

26 14 or 17 or 18 or 19 or 20 or 21 or 22 or 23 or 24 or 25

27 (physical and (exercise or activity or program or therapy)).mp.

28 physiotherapy.mp. or exp Physical Therapy/ or exp Physical Activity/

29 "manual therapy".mp.

30 27 or 28 or 29

31 26 and 30

32 ((multicomponent or multimodal or multifactorial or multidisciplinary or interdisciplinary or "mind-body") and (intervention* or treatment* or therap* or approach* or management)).mp.

33 exp Mind Body Therapy/

34 32 or 33

35 31 or 34

36 (older and (adult* or people or patient*)).mp.

37 "old age".mp. or exp Older Adulthood/ or exp Aging/

38 geriatric*.mp. or exp Geriatric Patients/ or exp Geriatrics/

39 (senior* or elderly).mp.

40 36 or 37 or 38 or 39

41 exp Pain/ or pain.mp. or exp Chronic Pain/ or "chronic pain".mp.

42 13 and 35 and 40 and 41 56

PubMed search strategy:

“telemedicine”[MeSH Terms] OR “telemedicine”[All Fields] OR “tele-medicine”[All Fields] OR “tele-health”[All Fields] OR “telehealth”[All Fields] OR “m-Health[All Fields]” OR “e-Health”[All Fields] OR “eHealth”[All Fields] OR “internet”[All Fields] OR “web”[All Fields] OR “online”[All Fields] OR “mobile”[All Fields] OR “app”[All Fields] OR “computer*”[All Fields] OR “technolog*”[All Fields] OR "virtual reality"[All Fields] OR "augmented reality"[All Fields]

AND

((((((((((“psychotherapy”[MeSH Terms] OR “psychotherapy”[All Fields] OR "psychosocial intervention"[MeSH Terms])) OR (((“psychological”[All Fields] OR “psychosocial”[All Fields])) AND ((“intervention*”[All Fields] OR “therap*”[All Fields] OR “treatment*”[All Fields])))) OR (("Relaxation Therapy"[MeSH Terms]))) OR ((“relaxation”[All Fields]) AND ((“technique*”[All Fields] OR “therap*”[All Fields])))) OR ((“Mindfulness”[MeSH Terms] OR “Mindfulness*”[All Fields] OR “psychoeducation”[All Fields] OR "psycho-education"[All Fields] OR "Behavior Therapy"[MeSH Terms] OR "cognitive behavioral therapy"[All Fields] OR "cognitive-behavioral therapy"[All Fields] OR "cognitive behavior therapy"[All Fields]))) OR ((“behavior”[All Fields]) AND ((“intervention*”[All Fields] OR “therap*”[All Fields] OR “treatment*”[All Fields])))) AND (((("physical therapy modalities"[MeSH Terms] OR “physiotherapy”[All Fields] OR “Exercise”[MeSH Terms])) OR ((“physical”[All Fields]) AND((“exercise” [All Fields] OR “activity” [All Fields] OR “program”[All Fields] OR “therapy”[All Fields])))) OR(("Musculoskeletal Manipulations"[MeSH Terms] OR "manual therapy"[All Fields] OR “rehabilitation”[All Fields])))) OR ((((“multicomponent”[All Fields] OR “multimodal”[All Fields] OR “multifactorial”[All Fields] OR “multidisciplinary”[All Fields] OR “interdisciplinary”[All Fields] OR "mind-body"[All Fields])) AND ((“intervention*”[All Fields] OR “treatment*”[All Fields] OR “therap*”[All Fields] OR “approach*”[All Fields] OR “management”[All Fields]))) OR (("Mind-Body Therapies"[MeSHTerms] OR "combined modality therapy"[MeSH Terms])))))

AND

(((“older”[All Fields]) AND ((“adult*”[All Fields] OR “people”[All Fields] OR “patient*”[All Fields]))) OR ((“Aged”[MeSHTerms] OR “elderly”[All Fields] OR “geriatric*”[All Fields] OR “senior*”[All Fields] OR "old age"[All Fields]))))

AND
((("chronic pain"[MeSH Terms] OR "chronic pain"[All Fields] OR "persistent pain"[All Fields] OR "long-term pain"[All Fields] OR “pain” [All Fields])))
Results: 3662

Web of Science Core Collection

Searches:
1: (telemedicine OR tele-medicine OR tele-health OR telehealth OR m-Health OR e-Health OR eHealth OR internet OR web OR online OR mobile OR app OR computer* OR technolog* OR "virtual reality" OR "augmented reality") (All Fields)

2: (ALL=((psychological OR psychosocial))) AND ALL=((intervention* OR therap* OR treatment*))

3: ALL=((psychoterapy OR psychoeducation OR "psycho-education" OR mindfulness*))

4: ALL=(("cognitive behavioral therapy" OR "cognitive-behavioral therapy" OR "cognitive behavior therapy"))

5: (ALL=(behavior)) AND ALL=((intervention* OR therap* OR treatment*))

6: (ALL=(relaxation)) AND ALL=((therap* OR techniq*))

7: #2 OR #3 OR #4 OR #5

8: (ALL=(physical)) AND ALL=((exercise OR activity OR program OR therapy))

9: ALL=((physiotherapy OR "manual therapy" OR "rehabilitation"))

10: #8 OR #9

11: #7 AND #10

12: (ALL=((multicomponent OR multimodal OR multifactorial OR multidisciplinary OR interdisciplinary OR "mind-body"))) AND ALL=((intervention* OR treatment* OR therap* OR approach* OR management))

13: #11 OR #12

14: (ALL=(older)) AND ALL=((adult* OR people OR patient*))

15: ALL=((eldery OR senior* OR geriatric* OR "old age"))

16: #14 OR #15

17: (ALL=((chronic OR persistent OR "long-term"))) AND ALL=(pain)

18: ALL=pain

19: #17 OR #18

20: #1 AND #13 AND #16 AND #19
Results: 621

Cochrane CENTRAL

#1 (telemedicine) OR (tele-health) OR (telehealth) OR (e-health) OR (ehealth) OR (m-health) OR (internet) OR (online) OR (web) OR (mobile) OR (app) OR (computer) OR (technolog*) OR (virtual reality) OR (augmented reality):ti,ab,kw

#2 (((((((psychological) OR (psychosocial)) AND ((intervention*) OR (therap*) OR (treatment*))) OR ((relaxation) AND ((therap*) OR (techniq*)))) AND ((psychoeducation) OR (psyho-education) OR (cognitive behavioral therapy) OR (cognitive-behavioral therapy) OR (cognitive behavior therapy) OR (mindfulness*))):ti,ab,kw OR ((behavior) AND ((intervention* OR therap* OR treatment*)))) AND ((((physical)) AND ((exercise) OR (activity) OR (program) OR (therapy))) OR ((physiotherapy) OR (manual therapy) OR (rehabilitation)))):ti,ab,kw OR (((multimodal) OR (multicomponent) OR (multidisciplinary) OR (interdisciplinary) OR (multifactorial) OR (mind-

body)) AND ((intervention*) OR (therap*) OR (treatment*) OR (program) OR (management*) OR (approach*))):ti,ab,kw

#3 ((older) AND ((adult*) OR (people) OR (patient*))) OR ((elderly) OR (seniors) OR (geriatric*) OR (aged) OR (aging) OR (old age)):ti,ab,kw

#4 (((chronic) OR (persistent) OR (long-term)) AND (pain)) OR (pain):ti,ab,kw

#5 #1 AND #2 AND #3 AND #4

Results: 484
