# Supplementary material for: eHealth-Integrated Psychosocial and Physical Interventions for Chronic Pain in Older Adults: Scoping Review
Source: J Med Internet Res. 2024 Jul 29;26:e55366. doi: 10.2196/55366 (PMC11319891; doi:10.2196/55366)
Supplement: Multimedia Appendix 2 [file jmir_v26i1e55366_app2.pdf]

Full-texts excluded and reasons for exclusion

**Full-text excluded (Studies identified via databases search)**	**Reason for exclusion**	
Heleno et al. [39]	*No e-Health intervention*	In-person intervention with no e-Health component
Stamm et al. [40]	*Trial registration*	Trial registry record concerning an intervention described in another paper by the same research group [65], which was already included in our review
Rybarczyk et al. [41]	*Wrong population*	The intervention targeted participants with chronic illnesses; only a low percentage of patients with chronic pain were included in the sample (27%), and the results were presented cumulatively without specific information on this sub-group of patients
Kelechi et al. [42]<br>Hernandez-Tejada et al. [43]		The intervention targeted participants with chronic illnesses; it was not explicitly indicated whether and to what extent participants had chronic pain; only less than half of the sample reported the use of pharmacological treatment for pain, hypothesizing that only a portion of participants presents pain
Suman et al. [44]		The target population consisted of general practitioners
Rini et al. [45]	*No multimodal intervention*	No physical component in the intervention; psychosocial component only
Madill et al. [46]		No physical component in the intervention; psychosocial component only
Allen et al. [47]		No psychosocial component in the intervention; physical component only (i.e., physical activity)
Wang et al. [48]		No psychosocial component in the intervention; physical component only (i.e., exercise)
Mace et al. [49]	*Secondary/preliminary studies evaluating the same interventions of the included papers*	Intervention already described in [62] (which was already included in our review)
Janevic et al. [50]		Intervention already described in [64] (which was already included in our review)
Fanning et al. [51]<br>Fanning et al. [52]<br>Fanning et al. [53]<br>Fanning et al. [54]		Intervention already described in [63] (which was already included in our review)
**Full-text excluded (Studies identified via forward citation tracking)**	**Reason for exclusion**	
Fanning et al. [55]	*Erratum of an included study*	Erratum of [63]
Mace et al. [56]	*Secondary studies evaluating the same intervention of included studies*	Intervention already described in [62] (which was already included in our review)
Vranceanu et al. [57]		Intervention already described in [62] (which was already included in our review)
Harris et al. [58]		Intervention already described in [59] (which was already included in our review)
