# Supplementary material for: eHealth-Integrated Psychosocial and Physical Interventions for Chronic Pain in Older Adults: Scoping Review
Source: J Med Internet Res. 2024 Jul 29;26:e55366. doi: 10.2196/55366 (PMC11319891; doi:10.2196/55366)
Supplement: Multimedia Appendix 3 [file jmir_v26i1e55366_app3.pdf]

Main characteristics of the included eHealth interventions

First Author	Main Aims	Main contents	Details	Duration; Format (group or individual); Administration modality (fully remotely or blended[a])	Main health professional delivering the intervention
Bennell et al. [59]	Self-management program to improve pain, physical function, and other bio-psycho-social variables	The Exercise Program (IG1): an individualized management plan, that included a progressive, muscle-strengthening home program; a physical activity plan, and other self-management strategies (e.g., activity pacing, pain coping skills, and sleep hygiene) plus education and behavioral counseling. The Diet and Exercise Program (IG2): all the exercise program components plus diet and additional dietary and behavioral resources	Both intervention groups were provided with six physiotherapist consultations via Zoom. The IG2 additionally received six dietitian consultations. Moreover, both groups had access to a website containing OA information	6 months; Individual format; Fully remotely	Physiotherapist, dietitian
Saraboon et al. [60]	Multifactorial intervention to improve health behavior and symptoms control	Multifactorial Intervention Program (MUFIPs). Components: health education program; weight-reduction/control program; quadriceps muscle exercise program; home visit program	In-person group workshop for 3 consecutive days (2 hours per day). Additionally, booklets and video compact discs for reference to OA knee information and positive health practice	8 weeks; Both Individual/group format; Blended	Nursing, nutritionist
Berman et al. [61]	Self-care management intervention for reducing pain intensity and interference and improving psychological variables	Mind-body self-care intervention. 6 core modules, including a selection of several exercises in each of the following areas: abdominal breathing, relaxation, writing about positive and difficult experiences, creative visual expression, and positive thinking. Additional contents: overview of complementary and alternative medicine, suggestions for how to communicate about pain, and general information about chronic pain	To access the website at least once a week and to complete the modules, being periodically monitored by a research assistant through email prompt	6 weeks; Individual format; Fully remotely	NS

Doorley et al. [62][b]	Mind-body program to improve physical, emotional, and cognitive functions in older adults with chronic pain and cognitive decline	The Active-Brains-Fitbit (AB-F). <u>Skills</u> to break the "disability spiral": physical activity, mind-body, pain and cognition awareness, cognitive functioning, and positive psychology skills	8 weekly (90-minute sessions) via Zoom. Participants were also provided with a Fitbit and an Actigraph accelerometer to monitor their daily activity	8 weeks; Group format; Fully remotely	Psychologist
Fanning et al. [63][c]	Behavioral intervention to improve pain, self-efficacy, functioning, and quality of life	The Mobile Health Intervention to Reduce Pain and Improve Health II (MORPH-II). <u>Contents</u>: goal review and revision, education on the importance of frequent physical activity and weight loss for pain management and quality of life, and practice in basic mindfulness skills	12 weekly 1-hour group sessions, which were held via teleconference software. Additionally, weekly diet and movement goals were tailored by health care professionals; specifically, participants aimed to reduce caloric intake and increase daily steps	12 weeks; Both Group/individual format; Fully remotely *(the original MORPH was blended with three in-presence meeting)*	Professional behavioral coach
Janevic et al. [64][d]	Self-management intervention to improve pain intensity and interference, and functioning	Positive Seniors using Technology to Engage in Pain Self-management (Positive STEPS) <u>Contents</u>: chronic pain mechanisms, goal setting, and relaxation exercises; increasing daily physical activity; music as pain medicine, make listening plan; making time pleasant activities; positive activities-life highlights or acts of kindness; positive activities-gratitude jar or savoring; review program takeaways. (*Based on STEPS-2 intervention. In Positive STEPS positive psychology activities have been added*)	7 weekly sessions including: watching web-based videos addressing specific pain management skills or positive activities and/or reading about these on a workbook; recording daily step count using an activity tracker; having a weekly 30-minutetelephone session with a designated community health worker	7 weeks; Individual format; Fully remotely	Community health worker
Stamm et al. [65]	Active Virtual reality exergame to improve pain intensity, functional capacities, fear-avoidance beliefs, and general physical and mental health	Interactive multimodal therapy. <u>12 exercises</u>: warm up (training of the upper and lower extremities); main part (strengthening of the abdominal and back muscles, core stability); cool down (stretching, progressive muscle relaxation exercise); psycho-educative units (topics:	Three 30-minute sessions per week in a laboratory setting. Participants performed interactive tasks using a VR handset and two controllers	4 weeks; Individual format; Blended	Physiotherapeutic supervision

		physiology of pain, pain management, stress management, everyday training)			
Godziuk et al. [66]	Behavioral health intervention to improve pain self-management and health-related quality of life.	<u>Core contents</u>: tips and meal planning guides; exercise instruction videos targeted for knee OA with progressions each week; videos on mindfulness and advice regarding self-care, motivation, and stress management, including goal setting activities. <u>Additional contents:</u> resources to self-manage positive health behaviors through tracking, monitoring, and encouragement; "Ask the Expert" (questions about nutrition, exercise, or mindfulness); nutrition consultation	OA core contents delivered through weekly emails; access to a web-based platform aimed at supporting general health and wellness behavior change; attendance at live webinar sessions held by health care professionals; one 30-minute one-to-one video/telephone consultation with a dietitian	12 weeks; Individual format; Fully remotely	Dietitian, psychologist, and kinesiologist
Pearson et al. [67]	Integrated rehabilitation program to improve pain self-management	The Enabling Self-management and Coping with Arthritic Pain through Exercise (ESCAPE-pain). <u>12 modules:</u> physical activity, exercise, goal setting, action plans, pacing, drug management, diet, home exercises, understanding pain, pain management, and relaxation. <u>Additional contents:</u> exercise videos; progress monitoring (input data and view graphical feedback on the amount of exercises, physical activity, mood, etc.); forum for group discussions	To test a prototype website, consisting of four external pages and four internal pages. They were then involved in semistructured "think aloud" interviews to get their opinions and experiences about the program	Duration NS; Individual format; Fully remotely	NS

[a]Blended: remote delivery plus additional in-person meetings.

[b]Secondary studies: [49,56,57].

[c]Secondary studies: [51–54].

[d]Secondary study: [50].

[e]IG: Intervention group.

[f]NS: Not specified.

[g]OA: Osteoarthritis.
