# Supplementary material for: eHealth-Integrated Psychosocial and Physical Interventions for Chronic Pain in Older Adults: Scoping Review
Source: J Med Internet Res. 2024 Jul 29;26:e55366. doi: 10.2196/55366 (PMC11319891; doi:10.2196/55366)
Supplement: Multimedia Appendix 4 [file jmir_v26i1e55366_app4.pdf]

Targeted outcomes, measures, and main results of the included studies

**First Author**	**Type of intervention group (IG) and control group (CG)**	**Measure tools**	**Outcomes**	**Results**		
Bennell et al. [59]	IG1 (Exercise Program) IG2 (Diet+Exercise Program) CG (Information only)			Difference in Change Between Groups (Baseline-6 months)		
				IG1>CG	IG2>CG	IG2>IG1
		Pain				
		Numeric Rating Scale	Pain intensity	Improvement[a] *P*=.01	Improvement[a] *P*<.001	Improvement[a] *P*=.005
		Psychological				
		Depression, Anxiety, and Stress Scale	Depression	NS[b]	Improvement *P*=.03	NS
			Anxiety	NS	NS	NS
			Stress	NS	NS	NS
		Physical				
		Western Ontario and McMaster Universities Osteoarthritis Index	Physical function	Improvement[a] *P*<.001	Improvement[a] *P*<.001	Improvement[a] *P*=.005
		Self-reported weight, measured in kilograms	Body weight	NS[c]	Improvement[a] *P*<.001	Improvement[a] *P*<.001
		Integrated				
		Assessment of Quality of Life 8-dimension instrument	QoL	Improvement *P* =.03	Improvement[a] *P*<.001	Improvement[a] *P*=.02
		Other				
		7-point Likert scale	Global change	Improvement[a] *P*<.001	Improvement[a] *P*<.001	Improvement[a] *P*<.001
		7-point Likert scale	Satisfaction with care	Improvement[a] *P*<.001	Improvement[a] *P*<.001	NS
		Yes/no question	Orthopedic appointments	NS	NS	NS
		5-point Likert scale	Unwilling to have knee surgery to willing	*P*=.001[a]	*P*<.001[a]	NS
			Willing to have knee surgery to unwilling	NS	*P*=.047[a]	NS
		Incidence	Total knee joint replacement and knee arthroscopy surgery		NA[d]	
Saraboon et al. [60]	IG (Multifactorial intervention programs) CG (Information only)			Difference between groups at post-test		
		Pain				
		Knee Severity Scale	Left knee		Improvement (IG>CG) *P*<.001	
			Right knee		Improvement (IG>CG) *P*<.001	
			Both knees		Improvement (IG>CG) *P*<.001	

		Psychological		
		Brief Illness Representation of OA Knee	Illness representation	Improvement (IG>CG)<br>*P*<.001
		Physical		
		Goniometer (Range of Movement)	Left knee flexion	Improvement (IG>CG)<br>*P*<.001
			Right knee flexion	Improvement (IG>CG)<br>*P*<.001
		Time Up and Go Test	Movement ability	Improvement (IG>CG)<br>*P*<.001
		CAMRY BR9807 personal spring weight scale	Weight	Improvement (IG>CG)<br>*P*<.001
		Integrated		
		Health Behavior Questionnaire	Health behavior related to OA knee	Improvement (IG>CG)<br>*P*<.001
		Other		
		OA Knowledge Test	OA knowledge	Improvement (IG>CG)<br>*P*<.001
Berman et al. [61]	IG (web-based mind-body intervention)<br>CG (Waiting list)			Within-group difference (Pre-post)
		Pain		
		Brief Short Pain Inventory-Short Form	Worst pain	Improvement (Both groups)<br>*P*=.01
			Least pain	Improvement (Both groups)<br>*P*=.05 (IG); *P*=.01 (CG)
			Average pain	Improvement (Both groups)<br>*P*=.01 (IG); *P*=.05 (CG)
			Average intensity	Improvement (Both groups)<br>*P*=.01 (IG); *P*<.001 (CG)
		Psychological		
		Pain Self-Efficacy Questionnaire	Self-efficacy	NS
		State-Trait Anxiety Inventory	Anxiety	NS
		Center for Epidemiologic Studies Short Depression Scale	Depression	NS
		Pain Awareness Questionnaire	Awareness of responses to pain	NS
			Confidence with managing pain	Improvement (IG only)<br>*P*<.001
		Integrated		
		Brief Short Pain Inventory-Short Form	Pain interference	Improvement (Both groups)<br>*P*=.01
		Other		
		Ad hoc survey	Satisfaction and use of the intervention	eHealth intervention was highly useful and user friendly
Doorley et al. [62]	IG (AB-F)<br>CG (Health Enhancement Program, HEP)			Within-group pre-post differences
		Pain		
		Numerical Rating Scale	Intensity at rest	NS
			Intensity with activity	Improvement (IG only)<br>*P*=.05; Cohen *d*[c]=1.0

Psychological		
Montreal Cognitive Assessment	Objective cognitive functioning	Improvement (IG only)<br>*P*=.05; Cohen *d* = 0.8
Everyday Cognition Scale	Self-reported cognitive functioning	NS
PROMIS anxiety	Anxiety	NS
PROMIS depression	Depression	NS
Pain Catastrophizing Scale	Pain catastrophizing	NS
Chronic Pain Acceptance Questionnaire	Pain acceptance	NS
Pain Self-Efficacy Questionnaire	Pain self-efficacy	NS
Measures of Current Status	Coping	NS
Tampa Kinesiophobia Scale	Kinesiophobia	NS
Cognitive and Affective Mindfulness Scale – Revised	Mindfulness skills	NS
Gratitude Questionnaire	Gratitude	NS
Self-Compassion Scale-Short Form	Self-compassion	NS
Physical		
Wrist-worn ActiGraph wGT3X-BT	Step count	Improvement (IG)<br>*P*=.04; Cohen *d*=0.4<br>Reduction (CG)<br>*P*=.02; Cohen *d*=0.3
6-min walk test via Timed Walk app	Timed walking performance	NS
Godin Leisure-Time Exercise Questionnaire	Physical activity intensity	Improvement (IG only)<br>*P*=.04; Cohen *d*=1.2
Integrated		
PROMIS Physical Function	Functional capacities	NS
World Health Organization Disability Assessment Schedule	Functional capacities	NS
PROMIS Emotional Support	Perceived emotional support	NS
UCLA Loneliness Scale	Loneliness	NS
Other		
Semi-structured exit interviews	Perceptions	Participants reported positive experiences and several benefits with the program.

		A priori set of benchmarks	Feasibility and acceptability	Both the programs met the criteria for "excellent" on nearly all of the a priori set benchmarks.
Fanning et al. [63]	IG (MORPH-II) CG (Low-contact condition)			Between-groups differences ANCOVAs
		Pain		
		PROMIS pain intensity scale	Pain intensity	NS
		Psychological		
		Psychological need satisfaction and need frustration scale	Autonomy Satisfaction	NS
			Autonomy Frustration	NS
			Relatedness Satisfaction	NS
			Relatedness Frustration	NS
			Competence Satisfaction	Improvement (IG>CG) *P*<.01; [f]η^2=0.22
			Competence Frustration	NS
		Physical		
		ActivPALTM 4 device	Physical activity (Daily steps)	Improvement (IG>CG) *P*=.02; η^2=0.23
			Average daily postural transitions	Improvement (IG > CG) *P*=.02; η^2=0.24
			Daily short sedentary time	NS
			Daily extended sedentary time	NS
		Integrated		
		PROMIS pain interference scale	Pain interference	NS
		Other		
		Attendance and retention	Feasibility	Retention rate 90.9% Session attendance on average 82.5%
		System Usability Scale	Acceptability	Average score = 77 ("good-to-excellent" usability); median score = 85 ("best imaginable" usability).
		Semi-structured interviews	Acceptability	Overall positive feedback on the program, described as "beneficial" and "life-changing".
Janevic et al. [64]	IG (Positive STEPS) CG (Waiting list)			Univariate ANOVA
		Pain		
		Numeric Rating Scale	Pain intensity	NA
		Psychological		
		Pain self-efficacy questionnaire	Pain self-efficacy	Improvement (IG>CG) *P*=.007; [g]η_p^2 =0.155
		Connor–Davidson Resilience Scale	Resilience	NS
		Physical		
		PROMIS-29	Physical functioning	NS
		Integrated		
		PROMIS-43	Pain interference	Improvement (IG>CG) *P*<.001; η_p^2=0.166
		PROMIS-29	Social participation	NS

		Other		
		7-point scale	Global impression of change	86% of IG vs. 25% of CG reported “better” or “much better” function.
		Proportion of completers	Retention	90%
		N of sessions completed and of completers of all videos	Engagement	Mean of 5.7 completed sessions. 95% of participants reported watching all program videos.
		Ad hoc questions	Acceptability	95% of participants strongly agreed/agreed that they increased their understanding of pain management and that the program helped them reach their pain management goals.
Stamm et al. [65]	IG (VR multimodal therapy)<br><br>CG (Conventional multimodal pain therapy)			Within-group pre-post differences
		Pain		
		Numeric Rating Scale	Pain intensity	NS
		Chronic Pain Grade Questionnaire	Pain severity	NA
		Psychological		
		Tampa Scale of Kinesiophobia	Kinesiophobia	NS
		Integrated		
		Hannover Functional Ability Questionnaire	Functional capacities	Improvement (IG only)<br>*P*=.03; r^h=0.67
		Health Survey SF-12 Scale	General physical health	NS
		Health Survey SF-12 mental	General mental health	Improvement (CG only)<br>*P*=.01; *r*=0.81
		Other		
		Technology Usage Inventory	Technology usage	Average score 19.09 points (higher degree of immersion).
		User Experience Questionnaire	User experience	Attractiveness and perspicuity were rated as “excellent’. Efficiency, dependability, and stimulation were rated as “good”. The originality of the VR solution was rated as “above average”.
Godziuk et al. [66]	NA			Within-group difference (Pre-post)
		Pain		
		Three-point scale	Mild severity	Improvement (3.8%)[i]
			Moderate severity	Improvement (13.2%)[i]
			Severe severity	Improvement (-17.0%)[i]
		Psycological		
		The Five Facet Mindfulness Questionnaire	Mindfulness	NS
		The PROMIS Self-Efficacy (SE)[j] for Managing Chronic Condition	SE for managing symptoms	Improvement *P*=.003
			SE for managing daily activities	Improvement *P*<.001
		The Arthritis Self-Efficacy Scale	Arthritis SE	NS

		Integrated		
		The 36-Item Short Form Health Survey	Physical functioning	Improvement $P<.001$
			Role limitations due to physical health	NS
			Role limitations due to emotional health	NS
			Energy/fatigue	NS
			Emotional well-being	NS
			Social functioning	NS
			Pain	Improvement $P=.02$
			General health	NS
		The Warwick-Edinburgh Mental Wellbeing Scale (WEMWBS)	Well-being	NS
		Other		
		Four-item Likert scale	Understanding of arthritis	Improvement $P<.001$
		Yes/no question	Interest in a TKA within the next year	Improvement $P<.001$
		Interview	Acceptability and engagement	Acceptability was demonstrated by positive perceptions of tailored intervention resources.
Pearson et al. [67]	NA	*Other*		
		Survey	Internet use and online activity	83% used the Internet; 69% described themselves as either very confident or confident when using the Internet, and 77% looked online for health information. 34% read a commentary or watched a video of someone else's experience of a health problem and 23% tracked a health issue.
		Focus group	Opinions	
		Semi-structured "think aloud" interviews	Acceptability and usability	Overall, the program was considered as a possible effective way to support the pain self-management.

[a]Result maintained at 12-months follow-up.
[b]NS: Not significant.
[c]Statistically significant result at 12-months follow-up.
[d]NA: Not applicable.
[e]Cohen *d*: Cohen's d effect size.
[f]η^2: Eta-squared effect size.
[g]η_p^2: Partial eta-squared effect size.
[h]*r*: *Mann*-Whitney-U-Test effect size.
[i]Difference in proportion.
[l]SE: Self-efficacy.
