# Supplementary material for: eHealth-Integrated Psychosocial and Physical Interventions for Chronic Pain in Older Adults: Scoping Review
Source: J Med Internet Res. 2024 Jul 29;26:e55366. doi: 10.2196/55366 (PMC11319891; doi:10.2196/55366)
Supplement: Multimedia Appendix 5 [file jmir_v26i1e55366_app5.pdf]

Assessment of studies' quality based on the QATSSD method

Study	Explicit Theoretical Framework	Statement of Aims/ Objectives in Main Body of Report	Clear Description of Research Setting	Evidence of Sample Size Considered in Terms of Analysis	Representative Sample of Target Group of a Reasonable Size	Description of Procedure for Data Collection	Rationale for Choice of Data-Collection Tool(s)	Detailed Recruitment Data	Statistical Assessment of Reliability and Validity of Measurement Tool(s)[a]	Fit Between Stated Research Question and Content of Data-Collection Tool[b]	Fit Between Stated Research Question and Method of Data Collection[a]	Fit Between Stated Research Question and Method of Analysis	Assessment of Reliability of Analytical Process[b]	Good Justification for Analytical Method Selected	Evidence of User Involvement in Design	Strengths and Limitations Critically Discussed	QATSDD Total Score
Bennell et al. [59]	1	3	3	3	3	3	2	3	1	NA	3	3		2	0	2	32
Saraboon et al. [60]	3	3	3	3	2	3	2	2	1	NA	2	3		1	0	2	30
Berman et al. [61]	1	3	3	1	2	3	3	3	3	NA	3	3		2	0	1	31
Doorley et al. [62]	2	3	3	3	2	3	3	3	3	NA[d]	3	3		2	3	3	39
Fanning et al. [63]	3	3	3	1	2	3	2	3	3	NA	3	3		3	2	3	37
Javenic et al. [64]	3	3	2	1	2	3	2	3	1	NA	3	3		3	3	3	35
Stamm et al. [65]	2	3	3	3	2	3	3	3	1	NA	3	3		3	2	3	37
Godziuk et al. [66]	2	3	3	3	2	3	2	3	1	3	3	3	3	3	0	3	35[c]
Pearson et al. [67]	2	3	3	1	2	3	3	3	0	3	2	3	3	3	3	2	34.5[c]
Mean value	2.1	3	2.9	2.1	2.1	3	2.4	2.9	1.6	3	2.8	3	3	2.4	1.4	2.4	34.5

Score: 0 = not at all; 1 = very slightly; 2 = moderately; 3 = complete.

[a]Applies only to quantitative studies.

[b]Applies only to qualitative studies.

[c]The mean score was calculated by subtracting from the total mean score the mean score of columns “Statistical assessment of reliability and validity of measurement tool(s)” and “Fit between stated research question and content of data collection tool, e.g., interview schedule” plus the mean score of columns “Fit between stated research question and method of analysis” and “Assessment of reliability of analytical process” since this study included both qualitative and quantitative aspects.
[d]NA: Not applicable.
